# The effect of alertness and attention on the modulation of the beta rhythm to tactile stimulation

**DOI:** 10.14814/phy2.14818

**Published:** 2021-06-26

**Authors:** Mia Illman, Kristina Laaksonen, Mia Liljeström, Harri Piitulainen, Nina Forss

**Affiliations:** ^1^ Department of Neuroscience and Biomedical Engineering Aalto University School of Science Espoo Finland; ^2^ Aalto NeuroImaging Aalto University School of Science Espoo Finland; ^3^ Faculty of Sport and Health Sciences University of Jyväskylä Jyväskylä Finland; ^4^ Department of Neurology Helsinki University Hospital and Clinical Neurosciences, Neurology University of Helsinki Helsinki Finland

**Keywords:** beta oscillation, event‐related desynchronization, event‐related synchronization, vigilance

## Abstract

Beta rhythm modulation has been used as a biomarker to reflect the functional state of the sensorimotor cortex in both healthy subjects and patients. Here, the effect of reduced alertness and active attention to the stimulus on beta rhythm modulation was investigated. Beta rhythm modulation to tactile stimulation of the index finger was recorded simultaneously with MEG and EEG in 23 healthy subjects (mean 23, range 19–35 years). The temporal spectral evolution method was used to obtain the peak amplitudes of beta suppression and rebound in three different conditions (neutral, snooze, and attention). Neither snooze nor attention to the stimulus affected significantly the strength of beta suppression nor rebound, although a decrease in suppression and rebound strength was observed in some subjects with a more pronounced decrease of alertness. The reduction of alertness correlated with the decrease of suppression strength both in MEG (left hemisphere r = 0.49; right hemisphere r = 0.49, *p < 0.05) and EEG (left hemisphere r = 0.43; right hemisphere r = 0.72, **p < 0.01). The results indicate that primary sensorimotor cortex beta suppression and rebound are not sensitive to slightly reduced alertness nor active attention to the stimulus at a group level. Hence, tactile stimulus‐induced beta modulation is a suitable tool for assessing the sensorimotor cortex function at a group level. However, subjects’ alertness should be maintained high during recordings to minimize individual variability.

## Graphical abstract

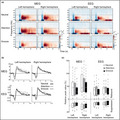

## 1 INTRODUCTION

The sensorimotor beta rhythm is mainly generated in the primary sensorimotor (SMI) cortex (Bardouille et al., 2019; Cheyne et al., 2003; Gaetz & Cheyne, 2006; Jurkiewicz et al., 2006), and it is known to be modulated by tactile (Cheyne et al., 2003; Gaetz & Cheyne, 2006; Illman et al., 2020; Parkkonen et al., 2015), electrical (Houdayer et al., 2006; Salenius et al., 1997; Salmelin & Hari, 1994), and proprioceptive stimulation (i.e., passive movement; Alegre et al., 2002; Illman et al., 2020; Parkkonen et al., 2015), as well as active movement (Cassim et al., 2000; Feige et al., 1996; Fry et al., 2016), action observation (Hari et al., 1998), or imagining motor action (Hari et al., 1998; Pfurtscheller et al., 2006; Schnitzler et al., 1997), and even by brief auditory or visual stimuli (Piitulainen et al., 2015). These stimuli and tasks, induce a rapid reduction (suppression or event‐related desynchronization, ERD) which is followed by a more delayed increase (rebound or event‐related synchronization, ERS) in the strength of rhythmic oscillations with respect to the baseline level (Pfurtscheller, 2001). It has been suggested that the suppression reflects cortical activation of the SMI cortex related to sensory afference and/or, movement preparation or initiation (Neuper et al., 2006; Pfurtscheller, 2001; Pfurtscheller & Lopes da Silva, 1999; Pfurtscheller et al., 1996). The rebound is thought to be associated with reduced excitability or active inhibition of the SMI cortex (Cassim et al., 2001; Chen et al., 1998; Engel & Fries, 2010; Gaetz et al., 2011; Pfurtscheller et al., 1996; Salmelin et al., 1995).

The beta rebound has been proposed to reflect the functional state of the SMI cortex in various neurological diseases such as stroke (Laaksonen et al., 2012; Parkkonen et al., 2017; Tang et al., 2020), schizophrenia (Brookes et al., 2015; Liddle et al., 2016), Parkinson's disease (Degardin et al., 2009; Hall et al., 2014; Vinding et al., 2019), and cerebral palsy (Demas et al., 2019; Pihko et al., 2014). However, patients are prone to changes in their alertness during MEG/EEG recordings, which may alter the oscillatory activity, and thus potentially affect the estimated cortical level of excitability. Alertness may easily decrease during MEG/EEG recordings in healthy individuals, and even more so in patients, for example, in acute stroke patients and patients suffering from cognitive disorders. In this study, we simulated clinical MEG and EEG measurement protocols to quantify the effect of alertness and active attention to the stimuli on the level of SMI beta rhythm modulation in healthy subjects. This new information is important for all future clinical and basic research studies that attempt to utilize the beta rhythm modulation to assess the SMI cortex function.

## 2 METHODS

### 2.1 Subjects

Twenty‐three healthy subjects (12 females, age 19–35, mean 23 ± 4 yrs) participated in the experiment. All subjects were right‐handed according to the Edinburgh Handedness Inventory (Oldfield, 1971).

The study was approved by the local ethics committee of Aalto University in accordance with the Declaration of Helsinki. Prior to the study, all participants signed written informed consent.

### 2.2 Stimuli and experimental design

Cerebral signals were recorded during three conditions to examine how the level of vigilance affects SMI cortex beta rhythm modulation. The conditions were selected from a practical point of view, as some patients may not be able to follow instructions during the MEG or EEG recordings. In the *neutral* condition, participants were fixating on a picture in front of them (size 12 × 15 cm, a distance of 2.2 m). The participants were instructed not to pay attention to the stimuli, and to think whatever comes into their mind. In the *attention* condition, the participants were fixating at the same picture as in the neutral condition, counting quietly in their mind the total number of the received tactile stimuli. The number of received stimuli was asked immediately after the attention task to ensure the subjects’ focus on the stimuli. During the *snooze* condition, the participants kept their eyes closed, without paying attention to the stimuli, and were allowed to fall asleep. The duration of all conditions was about nine to ten minutes and the conditions were measured in randomized order.

**FIGURE 1:**
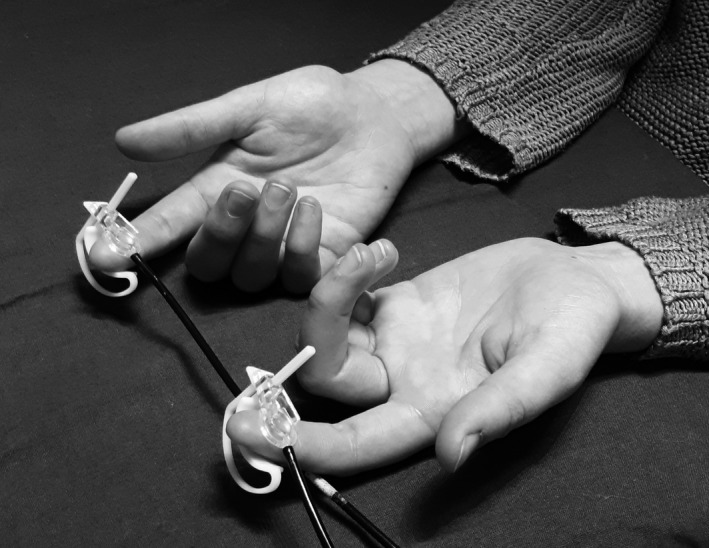
Tactile stimulus setup for beta rhythm modulation

Modulation of beta rhythm was induced by tactile stimuli that were delivered alternately to both index fingertips with an interstimulus interval (ISI) of 6 s for a given finger (3 s between right and left side stimulation). The stimuli were mechanically induced by pneumatic diaphragms driven by compressed air. The duration of the stimulus was 180 ms, peaking at 40 ms. During the stimulation periods, the participants held their hands relaxed on a pillow (Figure 1). Earplugs were used throughout the measurements to prevent possible stimulus‐induced noise artifacts.

### 2.3 Data acquisition

The simultaneous MEG and EEG measurements were carried out in a magnetically shielded room (Imedco AG, Hägendorf, Switzerland), with a 306‐channel (204 planar gradiometers, 102 magnetometers) whole‐head MEG system (Vectorview, Elekta Oy, Helsinki, Finland) at the MEG Core, Aalto NeuroImaging, Aalto University. Scalp EEG was recorded simultaneously with a MEG‐compatible 60‐channel EEG‐cap (ANT Neuro waveguard™original), where the Ag‐AgCl surface electrodes were placed according to the international 10–20 system. During the measurements, the participants were seated comfortably with their heads in the helmet‐shaped MEG sensor array. Prior to the measurement, five indicator coils were attached to the EEG‐cap (three to the forehead and two above the ears) to define the subject's head position with respect to the MEG sensors. The location of the indicator coils, anatomical landmarks (left and right preauricular points and nasion), and 100–200 additional points from the scalp surface, were determined with a 3‐D digitizer (Fastrak 3SF0002, Polhemus Navigator Sciences, Colchester, VT, USA). At the beginning of each measurement session, the head position inside the MEG helmet was measured with respect to the sensor array, and continuous head position tracking was monitored throughout the whole measurement. Eye movements were recorded with two vertical electrooculogram electrodes (EOG).

All data were recorded at a sampling frequency of 1000 Hz, and the MEG and EEG signals were band‐pass filtered to 0.1–330 Hz. The impedance of the EEG electrodes was verified to be below 5 kΩ prior to the recordings.

### 2.4 MEG and EEG signal processing

#### 2.4.1 Preprocessing

To improve the comparability of the different measurement conditions, MEG raw signals were transformed to the same average head‐coordinate system within each subject. The data was preprocessed off‐line using the temporal signal‐space‐separation method (tSSS) with head movement compensation (Taulu & Kajola, 2005; Taulu & Simola, 2006) implemented in the MaxFilter software (v2.2; Elekta Oy, Helsinki, Finland).

Further analyses of MEG and EEG data were done using MNE python 0.17 (Gramfort et al., 2013). The original EEG data (unipolar referential AFz) was re‐referenced with a common average reference overall electrodes (excluding bad channels). The average reference was chosen because our previous study indicated that this approach produced the highest signal‐to‐noise ratio and thus the strongest beta rhythm modulation (Illman et al., 2020). Artifacts related to eye blinks (two magnetometer and two gradiometer components) were removed with principal component analysis (PCA; Uusitalo & Ilmoniemi, 1997).

#### 2.4.2 Spectral analysis

Power spectral density (PSD) was calculated to observe changes in rhythmic brain oscillations at theta (4–7 Hz), alpha (8–12 Hz), and beta (13–25 Hz) frequencies during the different conditions. However, these PSDs do not represent spontaneous rhythmic brain oscillations, as they are affected by tactile stimulation. PSDs were computed for the neutral, attention, and snooze conditions by using the Welch method, with a sliding 2048‐point fast Fourier transform (FFT) with a non‐overlapping Hanning window. The peak power of theta, alpha, and beta frequencies was determined from the PSDs over the right and left SMI cortex and occipital area.

#### 2.4.3 Beta rhythm modulation

Time‐frequency representations (TFRs) were calculated to visualize changes in rhythmic activity in the three different conditions. TFRs for each subject were computed using a Morlet wavelet transformation in the frequency range of 2–40 Hz for a time window from –700 to 3200 ms with respect to stimulus onset (Tallon‐Baudry et al., 1997). Using wavelets, spectral and temporal resolution at different frequencies can be balanced by scaling the number of cycles by frequency. For this purpose, we set the number of cycles to f/2.

The strength of SMI cortex beta rhythm modulation was determined by computing the temporal spectral evolution (TSE) with respect to the onset of the tactile stimulus (Engemann & Gramfort, 2015; Hari & Salmelin, 1997). First, the pre‐processed raw data was bandpass filtered to 13–25 Hz. This 12‐Hz wide frequency band was chosen as our previous study (Illman et al., 2020) showed that individually selected 10 Hz frequency bands between 13 and 25 Hz (13–23 or 15–25 Hz) capture the strongest beta rhythm modulation. However, comparing individually selected frequency bands with common 13–25 Hz frequency band (capturing both the lower (β1) and higher (β2) beta bands) resulted in similar beta modulation curves. Therefore, we used in the present study the 13–25 Hz beta band for all the subjects, as standardized parameters particularly important in future clinical use. After filtering, interfering somatosensory evoked responses were subtracted from the raw data (David et al., 2006). A Hilbert transform was applied to the data to obtain the envelope signal, and the data were averaged with respect to stimulus onset. TSE curves were calculated from –500 to 3000 ms with respect to stimulus onset. The peak latencies and amplitudes of beta suppression and rebound were determined from the most representative MEG and EEG channels over the left and right SMI cortices. One or two channels with the strongest modulation were selected from both hemispheres (two channels were selected if the strongest suppression and rebound were seen over the different channels). Relative peak values (in %) of suppression (negative peak) and rebound (positive peak) were calculated with respect to the pre‐stimulus baseline (–500 to –100 ms).

### 2.5 Evaluation of alertness

#### 2.5.1 Questionnaire

The participants were asked to complete a questionnaire right after the MEG‐EEG measurement, to determine their overall alertness throughout the study. In the questionnaire, the participants evaluated their alertness subjectively during the three different conditions on a seven‐step Likert scale; 0 = Fell asleep, 1 = Fully tired, 2 = Moderately tired, 3 = Slightly tired, 4 = Slightly alert, 5 = Moderately alert, 6 = Fully alert.

#### 2.5.2 Sleep stage scoring

As the main purpose of the study was to clarify the effect of alertness on the modulation of the beta rhythm, the stage of alertness during the snooze condition was explored further. Sleep stages in the snooze condition were scored according to the AASM manual (American Academy of Sleep Medicine Manual for the Scoring of Sleep and Associated Events; Berry et al., 2012). The sleep stage was estimated from channels of the central, occipital and frontal regions, throughout the snooze condition in 30 s epochs. EOG channels were included in the sleep stage evaluation. Only Stage W, Stage N1, and Stage N2 were observed due to the short recording time. Stage W represents alert wakefulness to drowsiness (>50% of alpha rhythm and visible eye blinks), Stage N1 indicates sleep onset (vertex sharp waves, >50% of low voltage mixed frequency (LVMF) and slow eye movements), and Stage N2 light sleep (LVMF and K‐complexes or sleep spindles). Results are expressed in percentage with respect to the total snooze condition.

### 2.6 Statistical analysis

The non‐parametric Wilcoxon test was used to test differences in subjects’ self‐assessment of alertness between the neutral, attention, and snooze conditions. Normal distribution of relative peak values of beta suppression and rebound, and spectral peak amplitudes and frequencies, were tested with the Shapiro–Wilk test (IBM SPSS Statistics 26), resulting in a non‐normal distribution of the data. Statistical differences of suppression and rebound between the three different conditions were tested with the nonparametric Wilcoxon signed‐rank test. Spectral amplitudes of alpha, beta, and theta amplitudes were strongly skewed, and therefore the amplitudes were transformed logarithmically before the t‐test. In contrast, the nonparametric Wilcoxon signed‐rank test was used to test the frequencies, since the logarithmic correction had a minor effect on the normality of the data.

Correlation between the state of alertness (%) and the change in beta suppression/rebound strength in the neutral versus snooze conditions was tested with Spearman's correlation coefficient. The percentage decrease in alertness in the snooze condition was determined by summing the sleep stages N1 and N2 (weighting N2 by two).

A *p*‐value <0.05 was considered statistically significant in all tests. Bonferroni correction was used to correct the effect of multiple tests.

## 3 RESULTS

The measurements were performed successfully for all subjects and the quality of the obtained MEG and EEG data was good, despite a few poorly functioning MEG (2 channels) and EEG (1–3 channels) channels. In the attention condition, the subjects were highly focused on the stimuli, and all of them responded correctly to the number of stimuli at the end of the attention task. Most subjects (21 out of 23) had previous experience in participating in a MEG study, hence it was easy for them to relax in the snooze condition. For the TSE analysis, 95 ± 2 (mean ± SEM) averaged events were obtained in the neutral, 94 ± 1 in the attention, and 95 ± 1 in the snooze condition.

### 3.1 Level of alertness

**FIGURE 2:**
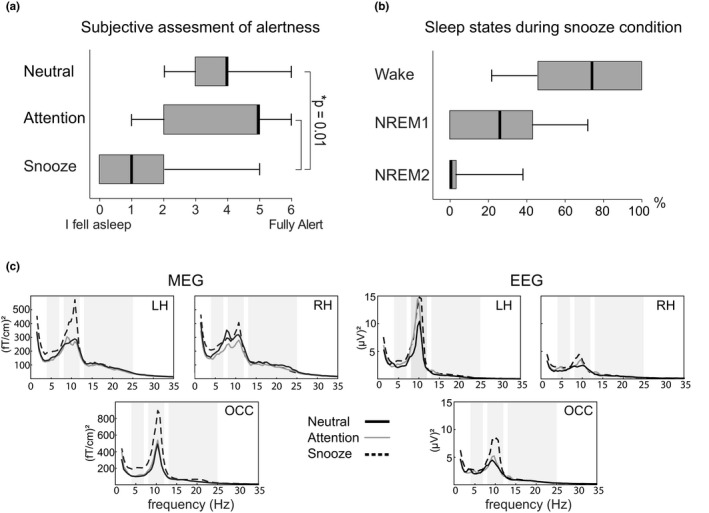
Assessment of participants’ alertness during the different conditions. (a) Participants’ subjective assessment of the alertness in the Neutral, Attention and Snooze conditions based on a questionnaire (Likert scale: 0 = I fell asleep, 1 = Fully tired, 2 = Moderately tired, 3 = Slightly tired, 4 = Slightly Alert, 5 = Moderately alert, 6 = Fully alert). (b) Sleep stage scores (in %) during the snooze condition according to the AASM manual (American Academy of Sleep Medicine Manual for Scoring of Sleep and Associated Events) based on the EEG recordings. (c) Grand averaged (n = 23) power spectra over left (LH) and right (RH) sensorimotor and occipital (OCC) areas during the Neutral, Attention, and Snooze conditions. The spectra have been calculated over the entire condition, including the changes of rhythmic activity caused by tactile stimulation

*Questionnaire*. According to the questionnaire, the participants felt clearly more tired (mean ± SD) in the snooze condition (1.6 ± 0.4) compared with the neutral (3.7 ± 0.3, *p* < 0.01), and attention condition (3.8 ± 0.4, *p* < 0.01); see Figure 2a.

*Sleep stage scores*. Figure 2b presents the subjects’ sleeping stages during the snooze condition. Due to the short measurement session, only three different stages of sleep were observed: Stage W, Stage N1, and Stage N2. On average, the subjects were in the a*wake stage* 70 ± 7%, *sleep stage N1* 26 ± 6%, and *sleep stage N2* 4 ± 2% of the total time of the snooze condition.

### 3.2 Peak power of theta, alpha, and beta frequencies during the different conditions

Figure 2c illustrates grand averaged (n = 23) power spectra over left and right SMI and occipital areas in the three conditions both in MEG and EEG. The peak power differed between the conditions in the theta and alpha frequencies, but not in the beta frequency band. The peak power over the occipital area was significantly stronger in the snooze vs. neutral conditions both in *the alpha* (MEG 1572 ± 266 vs. 659 ± 134 (fT/cm)^2^, **p < 0.01; EEG 32.6 ± 0.6 vs. 15.1 ± 3.4 (µV)^2^, *p < 0.05), and *theta frequency* band (MEG 238 ± 24 vs. 121 ± 16 (fT/cm)^2^, ***p < 0.001, EEG 3.9 ± 0.6 vs. 2.7 ± 0.6 (µV)^2^, **p < 0.01). The frequency of the peak power within the theta, alpha, and beta bands did not differ significantly between the conditions. Table 1 represents the peak power and frequency for each band and condition.

**TABLE 1:** Peak power and its frequency (mean ±SEM) for theta (4–7 Hz), alpha (8–12 Hz), and beta (14–25 Hz) bands during neutral, attention, and snooze conditions. The alpha frequency was determined for left and right sensorimotor (SMI) and occipital (OCC) areas, beta for left and right SMI areas, and theta for OCC area

	Theta		Alpha		Beta	
	OCC	Left SMI	Right SMI	OCC	Left SMI	Right SMI
MEG						
Peak frequency (Hz)						
Neutral condition	5.3 ± 0.1	10.3 ± 0.3	9.9 ± 0.3	10.2 ± 0.1	18.5 ± 0.6	18.0 ± 0.6
Attention condition	5.1 ± 0.2	10.3 ± 0.3	9.8 ± 0.3	10.0 ± 0.2	18.5 ± 0.7	18.1 ± 0.6
Snooze condition	5.4 ± 0.1	9.9 ± 0.3	9.7 ± 0.3	10.2 ± 0.2	17.9 ± 0.6	17.3 ± 0.5
Power (fT/cm)^2^						
Neutral condition	121 ± 16	496 ± 93	393 ± 60	659 ± 134	148 ± 31	138 ± 35
Attention condition	140 ± 20	397 ± 71	368 ± 66	828 ± 197	130 ± 30	119 ± 24
Snooze condition	238 ± 24	531 ± 71	531 ± 78	1572 ± 266	141 ± 30	128 ± 20
EEG						
Peak frequency (Hz)						
Neutral condition	5.2 ± 0.2	9.8 ± 0.3	9.8 ± 0.3	10.1 ± 0.2	17.2 ± 0.4	17.7 ± 0.5
Attention condition	5.2 ± 0.2	10.0 ± 0.3	9.9 ± 0.3	9.9 ± 0.2	17.5 ± 0.5	16.8 ± 0.5
Snooze condition	5.1 ± 0.2	10.0 ± 0.3	9.6 ± 0.2	10.0 ± 0.2	17.8 ± 0.6	17.5 ± 0.6
Power (µV)^2^						
Neutral condition	2.7 ± 0.6	6.0 ± 1.3	6.1 ± 1.4	15.1 ± 3.4	1.0 ± 0.2	1.1 ± 0.2
Attention condition	2.9 ± 0.6	6.5 ± 1.8	6.7 ± 1.9	22.6 ± 5.5	0.9 ± 0.2	1.1 ± 0.2
Snooze condition	3.9 ± 0.6	8.1 ± 1.8	8.6 ± 1.8	32.6 ± 6.0	1.1 ± 0.3	1.3 ± 0.3

### 3.3 Modulation of the beta rhythm

**FIGURE 3:**
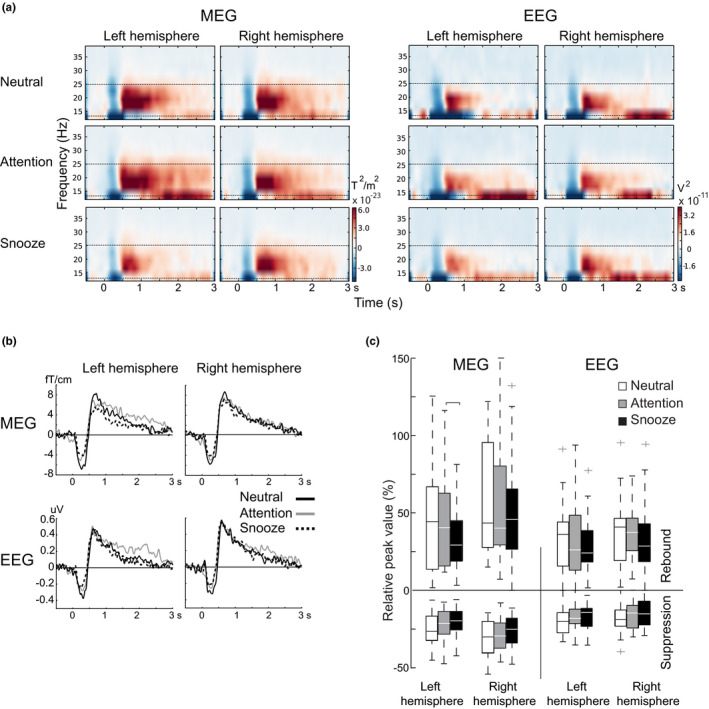
Modulation of beta rhythm during the Neutral, Attention, and Snooze conditions. (a) Grand averaged (n = 23) TFR images, and (b) TSE curves of the contralateral responses with respect to tactile stimulation in MEG and EEG. Zero point indicates the start of the stimulus. (c) Relative peak amplitudes (%) of beta suppression and rebound to tactile stimulation in the Neutral, Attention, and Snooze condition. The figure illustrates the responses of the contralateral hemisphere to the stimulated hand. 50% of the data points are inside the grey boxes and the white horizontal lines inside the boxes indicate the median values of beta suppression and rebound. Outliers of the data are shown by crosses

The modulation of the beta rhythm followed a similar pattern in all conditions both in MEG and EEG. An initial suppression of the beta rhythm peaked at around 300 ms after tactile stimulation, followed by a rebound at around 700–800 ms (Figure 3b). Beta rhythm suppression and rebound to tactile finger stimulation were observed bilaterally in sensors over the SMI cortices both in MEG and EEG. Suppression and rebound latencies did not differ significantly between the conditions (Table 2). As expected, the responses were clearly stronger in the contralateral hemisphere with respect to the stimulated hand, and therefore, the following results are provided only for the contralateral responses.

**TABLE 2:** Beta rhythm modulation strengths (relative to baseline) and latencies (mean ±SEM) in three different conditions for contra (CH) and ipsilateral (IH) hemispheres.

	Right stimulation				Left stimulation			
	MEG CH	EEG CH	MEG IH	EEG IH	MEG IH	EEG IH	MEG CH	EEG CH
Rebound								
Neutral								
Relative amplitude (%)	44 ± 7	35 ± 4	24 ± 4	16 ± 3	27 ± 4	23 ± 3	59 ± 8	37 ± 5
Peak latency (ms)	740 ± 33	759 ± 47	790 ± 38	773 ± 46	793 ± 37	728 ± 36	714 ± 30	667 ± 38
Absolute amplitude^a^	16.3 ± 4	0.89 ± 0.1					15.8 ± 3	0.91 ± 0.1
Attention								
Relative amplitude (%)	45 ± 7	33 ± 6	23 ± 4	16 ± 3	20 ± 4	18 ± 3	57 ± 8	39 ± 4
Peak latency (ms)	829 ± 48	761 ± 44	812 ± 46	808 ± 48	793 ± 47	737 ± 43	702 ± 32	682 ± 32
Absolute amplitude^a^	15.1 ± 3	0.79 ± 0.1					15.2 ± 3	0.90 ± 0.1
Snooze								
Relative amplitude (%)	34 ± 5	30 ± 4	24 ± 4	19 ± 3	22 ± 3	22 ± 3	50 ± 7	35 ± 4
Peak latency (ms)	773 ± 41	711 ± 41	729 ± 33	729 ± 38	801 ± 44	700 ± 28	703 ± 32	656 ± 26
Absolute amplitude^a^	10.7 ± 2	0.72 ± 0.1					13.4 ± 2	0.86 ± 0.1
Suppression								
Neutral								
Relative amplitude (%)	–25 ± 2	–20 ± 2	–25 ± 2	–19 ± 2	–19 ± 2	–16 ± 2	–31 ± 2	–18 ± 2
Peak latency (ms)	298 ± 15	326 ± 20	320 ± 13	327 ± 21	343 ± 26	321 ± 17	293 ± 20	288 ± 19
Absolute amplitude^a^	–10.4 ± 2	–0.58 ± 0.1					–9.6 ± 2	−0.51 ± 0.1
Attention								
Relative amplitude (%)	–22 ± 2	–17 ± 2	–23 ± 2	–17 ± 1	–20 ± 2	–16 ± 2	–29 ± 2	–17 ± 2
Peak latency (ms)	269 ± 20	266 ± 19	314 ± 20	302 ± 20	318 ± 19	275 ± 26	255 ± 22	272 ± 19
Absolute amplitude^a^	–7.9 ± 1	–0.47 ± 0.1					–8.7 ± 2	–0.46 ± 0.1
Snooze								
Relative amplitude (%)	–20 ± 2	–17 ± 2	–21 ± 2	–13 ± 2	–15 ± 2	–12 ± 1	–26 ± 2	–15 ± 2
Peak latency (ms)	235 ± 17	270 ± 21	275 ± 17	313 ± 24	300 ± 22	329 ± 20	250 ± 18	275 ± 218
Absolute amplitude^a^	–6.3 ± 1	–0.43 ± 0.1					–7.5 ± 1	–0.38 ± 0.1

^a^ MEG, fT/cm; EEG, uV.

#### 3.3.1 Time‐frequency representation

Figure 3a illustrates the grand average (n = 23) strength and temporal behavior of the beta rhythm with respect to stimulus onset in all three different conditions. Both in MEG and EEG, the temporal behavior of the beta suppression and rebound was similar in all three conditions. However, the strengths of suppression and rebound appear slightly diminished in the snooze compared to the attention and neutral conditions, especially in MEG. In the attention condition, the rebound appeared somewhat prolonged compared to the neutral and snooze conditions, especially in the left hemisphere.

#### 3.3.2 Beta rhythm modulation

Figure 3b illustrates the grand average (n = 23) TSE curves over the contralateral SMI cortex with respect to the stimulated hand during the neutral, attention, and snooze conditions. Figure 3c shows that the contralateral relative peak strengths of beta suppression and rebound did not differ significantly between the conditions. In MEG, the rebound appeared to be lower in the snooze condition compared to the neutral condition (34 ± 5 vs. 44 ± 7 in the left and 50 ± 7 vs. 59 ± 8 right hemisphere), although the difference was not significant. Table 2 shows the mean strengths of the beta rhythm modulation.

**FIGURE 4:**
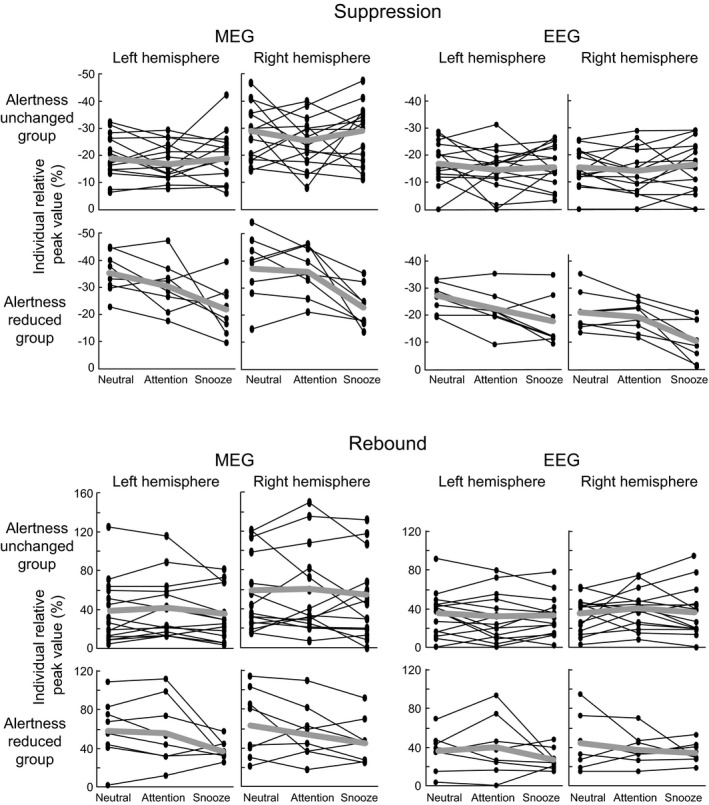
Individual relative peak strengths of the beta suppressions and rebounds for all subjects in the Neutral, Attention, and Snooze conditions. Subjects are divided into two categories; “Alertness reduced” (n = 8) and “Alertness unchanged” group (n = 15), based on sleep state scores and alertness self‐assessment in the snooze condition. Subjects with more than 35% of sleep stages N1 and N2 and who also reported falling asleep during the snooze condition according to self‐assessment, were included in the “Alertness reduced” group

**FIGURE 5:**
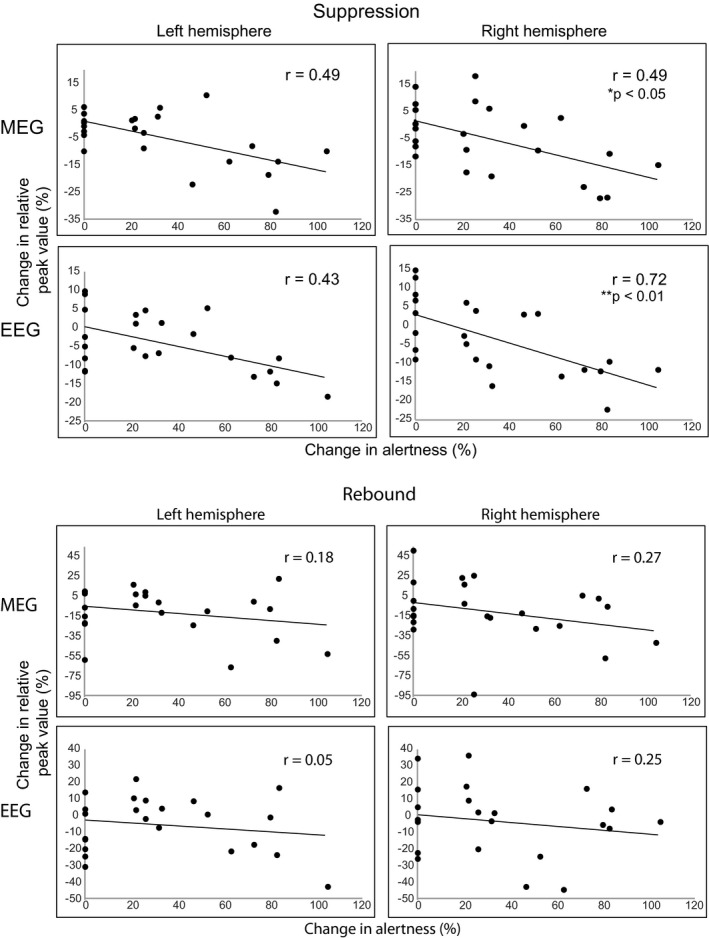
Correlations between the level of alertness in the snooze condition and changes in the strength of beta suppression and rebound between the neutral and snooze condition. The change in alertness is described by the percentage of summed sleep stages N1 and N2 (N2 weighted by two)

Figure 4 shows the individual relative peak strengths of suppression and rebound for all subjects. The subjects were divided into two groups "Alertness unchanged" and "Alertness decreased", indicating a pronounced reduction of the suppression and rebound in the snooze condition in the subjects with decreased alertness (n = 8) compared to subjects whose alertness did not change remarkably. However, the individual variation between different situations is worthy to note. Furthermore, Figure 5 illustrates the correlations between the level of alertness during the snooze condition and the change in suppression and rebound strength between the neutral and snooze conditions. Reduced alertness correlated significantly with the reduction of suppression strength in the right hemisphere in all subjects both in MEG r = 0.49, *p < 0.05 and EEG right hemisphere r = 0.72, **p < 0.01, hence, the larger the change in alertness the stronger the reduction in suppression strength. In contrast, no correlations between changes in alertness and changes in rebound strengths were observed.

#### 3.3.3 Baseline beta power

**FIGURE 6:**
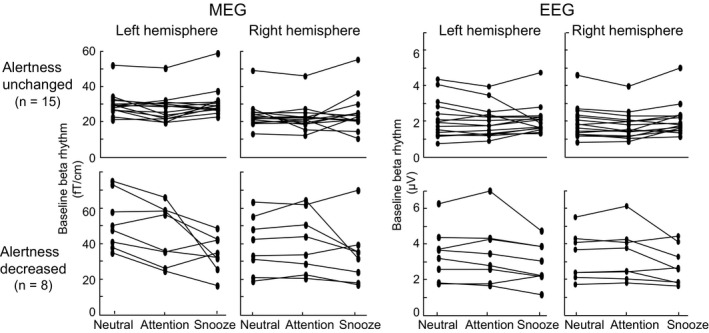
Baseline beta power in TSE for all subjects during the Neutral, Attention, and Snooze conditions. Subjects in the “Alertness reduced” group had over 35% of sleep stages N1 and N2 and they also reported to fall asleep according to self‐assessment during the snooze condition

Table 3 shows mean (± SEM) baseline beta power values from –500 to –100 ms during the neutral, attention, and snooze conditions. The baseline beta power remains stable between different conditions, with exception of the left hemisphere in MEG, which showed a significant difference between the neutral and attention conditions (*p* = 0.02). Figure 6 illustrates all subjects’ individual baseline changes in different conditions. The subjects are further divided into the "Alertness unchanged" and "Alertness decreased" groups, showing that baseline changes are larger in the "Alertness decreased" group in MEG.

**TABLE 3:** Baseline (–500 to −100 ms) beta power values (mean ±SEM) from TSE curves over left (LH) and right (RH) sensorimotor cortex during the neutral, attention, and snooze conditions in MEG and EEG

MEG (fT/cm)	LH	RH	EEG (µV)	LH	RH
Neutral	37.7 ± 3	29.2 ± 3	Neutral	2.6 ± 0.3	2.4 ± 0.3
Attention	33.8 ± 3*	28.9 ± 3	Attention	2.5 ± 0.3	2.4 ± 0.3
Snooze	31.9 ± 3	28.0 ± 2	Snooze	2.4 ± 0.2	2.4 ± 0.2

* *p* < 0.05.

As the baseline values showed some differences between the conditions, the beta suppression and rebound strengths were analyzed from absolute values (Table 2). In line with the results obtained from the analysis of relative peak strengths, the absolute suppression and rebound strengths did not show significant differences between the conditions.

In summary, at the group level, the strength of suppression and rebound did not differ between the three conditions. However, there was a weak correlation between reductions in alertness and beta suppression strength.

## 4 DISCUSSION

To our knowledge, this is the first study investigating the effect of change in alertness on beta rhythm modulation. At the group level, reduced alertness or active attention to the received tactile somatosensory stimulus did not significantly affect the SMI beta rhythm modulation. However, in some subjects with a pronounced reduction in alertness, a remarkable decrease of suppression and rebound strength was observed. Moreover, reduced alertness correlated with changes in suppression strength, indicating that at the individual level changes in alertness may affect the strength of rhythmic modulation. This is an important topic especially as the beta modulation has been proposed to serve as a biomarker of the functional state of the SMI cortex in several neurological conditions, where the alertness may often be reduced.

### 4.1 Power spectra

Spontaneous rhythmic brain activity changes remarkably between stages of alertness and from a sleep stage to another. Spontaneous alpha and beta rhythms are predominant during wakefulness. When a person enters into a light sleep, the alpha rhythm is reduced, while slower rhythmic activity (theta 4–7 Hz and delta 1–4 Hz) enhances (Broughton & Hasan, 1995), predominantly in the frontal cortex (Marzano et al., 2013). Our observed increase in theta rhythm strength in the snooze condition confirms that our results are reflecting well the effect of reduced alertness on rhythmic brain activity. In contrast, the increased alpha rhythm during the snooze condition is most likely due to the well‐known effect of eyes closure at the beginning of the snooze condition before falling asleep. MEG measurements in a quiet environment of the magnetically shielded room may cause the experience of boredom, sustained attention, or even mental fatigue, which can affect a variety of brain rhythms (Lal & Craig, 2001; Langner & Eickhoff, 2013; Shigihara et al., 2013; Tanaka et al., 2012, 2014). Low vigilance has been described to reduce the power of spontaneous beta oscillations in the SMI cortex (Belyavin & Wright, 1987), but such changes in the beta rhythm were not observed in the current study. However, in the present study, the actual spontaneous data were not recorded as the data was contaminated with the tactile stimuli.

Natural inter‐individual variation of beta rhythm peak frequency and strength is expansive, and heritability regulated (Salmelin & Hari, 1994; Smit et al., 2005). The circadian regulation has an effect on the spontaneous beta power, which has been described to be weakest in the morning and increasing towards the afternoon (Cacot et al., 1995; Toth et al., 2007). Such circadian changes have also been described to have an effect on the modulation of the beta power, primarily on the beta suppression (Wilson et al., 2014). To control for circadian changes in rhythmic activity, in the present study, the measurements were recorded between 11 am and 5 pm, a time span, where the rhythm is supposed to be strongest.

### 4.2 Effects of alertness on the modulation of the SMI beta rhythm

At the group level, reduced alertness did not significantly affect the strength of SMI beta rhythm modulation. Although reductions in suppression and rebound strengths were observed in some subjects with markedly reduced alertness, changes in the opposite directions were also observed, and thus the changes were not consistent across the examined subjects. Inter‐individual variation in the level of alertness may have had an effect on the large variability of the results. Furthermore, the eyes closure in the snooze condition may have affected the results. However, an earlier study indicated that eye closure alone does not alter the strength of beta rhythm modulation (Rimbert et al., 2018). The correlation analysis between changes in alertness and changes in beta modulation indicated that decreased alertness affected mainly the strength of beta suppression but not rebound. This is an interesting finding as, in contrast, the beta rebound has previously shown to be more sensitive to changes in stimulus modality (such as tactile vs. electrical stimulus or speed and range of movement Cassim et al., 2000; Fry et al., 2016; Houdayer et al., 2006; Parkkonen et al., 2015; Pfurtscheller et al., 1998; Salenius et al., 1997; Salmelin & Hari, 1994) than the suppression. The suppression and rebound are thought to arise from separate neuronal populations, and to have distinct functional roles (Cassim et al., 2000; Chen et al., 1998; Hall et al., 2011; Jurkiewicz et al., 2006; Salmelin et al., 1995). The current study is in line with these earlier findings as the suppression and rebound appeared to respond to changes in alertness in distinct ways.

Based on the results, decreased alertness does not significantly affect the strength of beta modulation, especially the beta rebound, at the group level. These findings support the reliability of group‐level findings of changes in beta suppression/rebound, that is, in different clinical conditions. Especially the minimal effect of reduced alertness on the strength of beta rebound is important, as the beta rebound has been suggested as a biomarker of the functional state of the SMI cortex after stroke (Laaksonen et al., 2012; Parkkonen et al., 2017, 2018; Tang et al., 2020). However, at the individual level, alterations in alertness may affect beta rhythm modulation, especially beta suppression, which should be taken into account in longitudinal experiments to avoid misinterpretations.

In the present study, the level of alertness was assessed in three different ways, which all confirmed a decrease in alertness in the snooze condition. Although drowsiness of healthy subjects is not equivalent to reduced alertness of an acutely ill patient, the results clearly indicate that beta modulation is suitable as a biomarker also in acute patients. In our experience, only some acute stroke patients had challenges in maintaining alertness during measurement. Taken together, the possible effect of decreased alertness on beta modulation is not significant at the group level. However, it is advisable to monitor changes in the level of alertness during measurements and to encourage the study subjects to be eyes open and keep their vigilance as good as possible. Moreover, it is recommended that measurements are taken at the time of day when subjects are most alert.

### 4.3 Effects of active attention to the stimulus on the modulation of the SMI rhythms

In general, attention to a sensory stimulus has been shown to alter rhythmic brain activity. Visual alpha is most extensively studied, and it has been shown to reduce brain regions primarily engaged in visual tasks and enhance in regions that are less involved (Van Diepen et al., 2019; Foxe & Snyder, 2011; Klimesch, 2012; Palva & Palva, 2007). These spatial modulations in alpha power are thought to reflect a general mechanism of attentional gating in the cortical processing involved and inhibition in various other brain regions. Much less is known about the effects of attention on the beta rhythm of the Rolandic sensorimotor cortex. Beta band power has shown to be negatively correlated with the dorsal attention network including the sensorimotor area (Sadaghiani et al., 2010). Beta rhythm decreases during the attention task associated with multisensory stimuli (Friese et al., 2016; Misselhorn et al., 2019), and to increase in relation to faster reaction time (i.e., increased alertness) to visual stimuli (Kaminski et al., 2012), as well as during enhanced attention to tactile stimuli (Bardouille et al., 2010). More focused attention to a tactile stimulus either increased (Bardouille et al., 2010; Dockstader et al., 2010) or decreased (Bauer et al., 2006) the strength of beta suppression and rebound. The expectation of an upcoming tactile stimulus has been shown to produce the suppression prior to the stimulus (van Ede et al., 2010), however, the attention‐related beta suppression was not seen prior to the stimulus onset in our study. These varying results indicate that active attention affects the sensorimotor cortex beta rhythm, but the large variety of stimuli and tasks used in the studies may have different impacts on the beta rhythm. The simple attention task used in the present study additionally showed a prolonged beta rebound in the left hemisphere, which may reflect that vigilance is more regulated in the left hemisphere, as has also been shown in a previous study (Kim et al., 2017). However, the current study indicates that the unwanted attention to the regularly repetitive tactile stimulation has only inconsistent minor changes on the beta rhythm modulation, and thus the unfavorable behavior of subjects does not distort the results.

### 4.4 Baseline beta power

In line with some earlier studies (Anderson & Ding, 2011; van Ede et al., 2010, 2011; Jones et al., 2010), a slightly decreased pre‐stimulus baseline was observed in the attention condition compared to the other conditions, which may have an effect on the relative suppression and rebound strengths. However, the difference was significant only in the left hemisphere. As any baseline differences between different conditions may affect the results, the suppression and rebound strengths were revised from the absolute strengths (as done, e.g., in Muthukumaraswamy et al., 2013). The absolute modulation strengths did not differ between the conditions in line with the results obtained from the relative values. Therefore, the effect of the baseline power appeared to be negligible, and the baseline normalized relative values are appropriate also for clinical use.

## 5 CONCLUSION

The present study simulated the measurement protocol of acute stroke patients to study the effect of alertness and attention to the stimulus on SMI beta modulation. Neither reduced alertness nor active attention to the stimulus had a significant effect on the strength of suppression or rebound of the beta rhythm at the group level. This important observation shows that minor changes in alertness do not significantly affect the results of beta modulation studies. However, the effect of alertness on beta modulation was individual and may be stronger in some subjects and patients. Thus, individual results should be evaluated with caution. It is also important to minimize the effects of changes in alertness in longitudinal patient studies, where the risk of changes in alertness can be substantial between measurements.

## CONFLICT OF INTEREST

No conflicts of interest, financial or otherwise, are declared by authors. The authors declare that they have no known competing financial interests or personal relationships that could have appeared to influence the work reported in this paper.

## AUTHOR CONTRIBUTIONS

MI, KL, HP conceptualized and designed the study. MI performed the experiments and analyze data. MI drafted the manuscript. All authors edited and revised the manuscript, and approved the final version of the manuscript.
